# Performances of the hybrid between CyCa nucleocytplasmic hybrid fish and scattered mirror carp in different culture environments

**DOI:** 10.1038/srep46329

**Published:** 2017-04-12

**Authors:** Xiangjiang Liu, Hongwei Liang, Zhong Li, Yongjun Liang, Cuiyun Lu, Chitao Li, Yumei Chang, Guiwei Zou, Guangfu Hu

**Affiliations:** 1 College of Fisheries, Key Laboratory of Freshwater Animal Breeding, Ministry of Agriculture, Freshwater Aquaculture Collaborative Innovation Center of Hubei Province, Huazhong Agricultural University, Wuhan, 430070, China; 2 Yangtze River Fisheries Research Institute, The Chinese Academy of Fisheries Sciences, Wuhan, 430223, China; 3 Beijing Key Laboratory of fishery Biotechnology, Beijing Fisheries Research Institute, Beijing, 100068, China; 4 Key Laboratory of Freshwater Aquatic Biotechnology and Breeding, Ministry of Agriculture, Heilongjiang Fisheries Research Institute, Chinese Academy of Fishery Sciences, Harbin 150070, China

## Abstract

To improve the performance of growth traits and survival in common carp, CyCa nucleocytoplasmic hybrid fish (C) was used as parental fish for hybridization with Russian scattered mirror carp (R). Performances in morphological characters, growth traits and survival rate were compared among the purebreds (CC & RR) and crossbreds (RC & CR) at different time period in solitary and communal rearing system, respectively. The results demonstrated that both RC and CR crossbreds inherited the grey skin color type from the mirror carp, and got the full-scale pattern from the CyCa nucleocytoplasmic hybrid fish, which suggested that the grey color dominated to red color and full-scale dominated to scattered scale. With respect to yield, the RC crossbreds perform quite great compared to the RR and CC purebreds because they have quite high growth and survival rate. In contrast to RC crossbreds, the CR crossbreds performed poorly in growth traits, together with that crosses where scattered mirror carp was used as mother (RC and RR) achieved the greatest performance for all growth traits, suggested that the maternal influence also displayed an important role in growth traits. These results indicated that the RC crossbreds will be a potential carp variety for commercial production.

Heterosis was first reported by Shull in 1908 to describe the phenomenon of a hybrid offspring with enhanced viability and developmental rates compared with its parents^1^. Although the genetic mechanism of heterosis was only partly understood^2^,^3^,^4^, crossbreeding has been widely applied for the genetic improvement of animals and plants^5^,^6^,^7^,^8^. In teleost, crossbreeding was also widely used to increase growth rate, manipulate sex ratios, produced sterile fish and to improve flesh quality, disease resistance and environmental tolerance^9^,^10^. In common carp, systematic crossbreeding has been performed, and the significance of heterosis for growth rate and other traits (survival and disease tolerance) among crosses of wild and domesticated European, Russian, Chinese and Japanese strains have also been repeatedly reported^11^. Besides over all, the gain achieved through crossbreeding may also be equivalent to several generation of selection, and can simultaneously improve other important traits such as growth rate and disease resistance^12^, so it is a very simple and cost-effective strategy for genetic improvement and fish breeding in aquaculture industries.

In China, common carp (*Cyprinus carpio L*.) has been cultivated in ponds as a food fish for more than 2500 years^13^. During this time, the common carp has developed a great number of genetically different varieties or strains as a result of geographic isolation, accumulations of mutations and selection pressures^11^. Of these, Hebao red carp (*Cyprinus carpio* var. *wuyuanensis*) and Xingguo red carp (*Cyprinus carpio* var. *singuonensis*) are two traditional domestic varieties, which have been cultivated for about 1300 and 800 years in China, respectively^14^. In addition, for need of decorative and genetic improvement, scattered mirror carp was also introduced into China from Russia. The three strains were the representative varieties and many commercial hybrids of common carp in China were produced by using them as parental lines, such as Feng carp (Xingguo red carp♀ × Scattered mirror carp♂), Lotus carp (Scattered mirror carp♀ × Xingguo red carp♂), Heyuan carp (Hebao red carp♀ × *C. Carpio* var. *yuankiang*♂) and Tri-crossed carp (Heyuan carp♀ × Scattered mirror carp♂).

Except these traditional varieties, Chinese scientists also succeeded in generation of CyCa nucleocytoplasmic hybrid fish to study the interaction between nucleus and cytoplasm^15^,^16^. In their studies, the blastulae nucleus of Hebao red carp (*Cyprinus carpio var. wuyuanesis*) was transplanted into the unfertilized enucleated Yangtze River crucian carp (*Carassius auatus*) egg. After removing the egg capsule with forceps, the nucleus of the crucian egg was picked out with a glass microneedle at the point immediately underneath the very small polar body, to obtain an enucleated crucian carp egg. Nuclei from the middle or late blastula stage of carp embryos were transplanted into the enucleated eggs of crucian. Each egg received one nucleus at the center of the blastoderm of the animal hemisphere. Only 29 transplanted eggs (about 3.2%) were developed to adult hybrid fish, while most of the others were arrested at various embryonic stages. The morphological characteristics of the CyCa nucleocytoplasmic hybrid fish became distinguishable when they reached adulthood. Some morphological features were inherited from the nucleus donor fish such as bards and pharyngeal teeth, some seemed to come from the cytoplasm host fish such as the number of vertebrae and some were intermediates such as the number of scales along the lateral line, which suggested that both nucleus and cytoplasm can influence the expression of genetic information on the hybrid fish. Through two years of careful breeding, some of the male and female CyCa nucleocytoplasmic hybrid fish developed to maturity. Through artificial insemination between these fish, all the eggs were fertilized and developed into adult fish. The growth rate in both F2 and F3 generation of CyCa nucleocytoplasmic hybrid fish is 22% higher than Hebao red carp which is their nucleus donor. Furthermore, biochemical analysis showed that the protein content of the muscle of CyCa nucleocytoplasmic hybrid fish is 3.78% higher and the fat content is 5.58% lower than that of red common carp, respectively^17^. These results suggested that CyCa nucleocytoplasmic hybrid fish might be a better parent to produce hybrids.

In this study, a diallel cross between CyCa nucleocytoplasmic hybrid fish and scattered scale mirror carp was carried out, the growth traits and survival rate were compared among the two parent lines and their reciprocal cross lines under both solitary and communal rearing conditions at different time period. Our objectives were (i) to investigate the presence of heterosis between CyCa nucleocytoplasmic hybrid fish and scattered mirror carp, (ii) to estimate heterosis in different rearing conditions. Moreover, by using the correlation analysis, we also estimate the contribution of growth-related traits to body weight, which will provide useful information for better understanding the relationships among growth-related traits for further selective breeding of crossbred and purebred lines.

## Results

### Morphological characteristics of the purebreds and crossbreds

**Figure 1:**
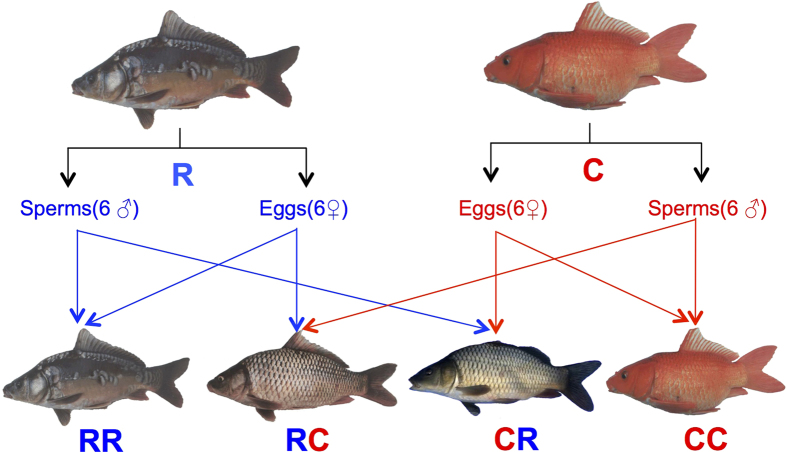
The 2 × 2 diallel cross design by pairwise mating between different individuals within the same subspecies and between the two subspecies of common carp. R and C represent two parental populations of Russian scattered mirror carp and CyCa nucleocytoplasmic hybrid fish, RR and CC represent two purebred offspring produced by crosses between different individuals within the same subspecies, and RC and CR represent two crossbred offspring produced by crosses between two subspecies, respectively.

As shown in Fig. 1, CyCa nucleocytoplamic hybrid fish (C♀ × C♂, CC) purebred offspring displayed red skin color and full-scale pattern, whereas the Russian scattered mirror carp (R♀ × R♂, RR) purebreds showed a typical grey scattered scale pattern. Interestingly, their reciprocal crosses lines (R♀ × C♂, RC; and C♀ × R♂, CR) inherited the grey skin color from the scattered mirror carp, and got the full-scale pattern from the CyCa nucleocytoplasmic hybrid fish. Therefore, it is easy to distinguish purebred and crossbred offspring by their skin color and scale pattern (Supplementary Fig. S1).

### Performances of four groups under solitary rearing condition

Performance traits of two purebreds and two crossbreds under solitary rearing condition from day 62 to 709 were presented in Table 1. The mean body weight in RC crossbreds and RR purebreds were significantly greater than that of other two mating combinations (CC purebreds and CR crossbreds) on day 152, 181, 213 and 709, respectively (P < 0.05). The mean body weight of RC crossbreds was always higher than that of RR purebreds during the whole study period, but this difference was significantly observed at 213 and 709 days old (P < 0.05). During the whole grow-out stage, the mean value of body weight in RC crossbreds was the highest, but that of the CC purebreds was the lowest. The mean value of standard length in RC crossbreds was always lower than that of RR purebreds during the whole study period, and the differences were significantly found at 123, 152 and 181 days old (P < 0.05). The highest mean values for standard length were always observed in the RR purebreds during the whole study periods. The mean value of standard length in CC purebreds was significantly shorter than that of other three mating combinations since day 123 (P < 0.05). On the day 152, 181, 213 and 709, pre-dorsal height (PDH) in RC crossbreds was significantly larger than that of the other three mating combination (P < 0.05) following in the order that: RC < RR < CR < CC. Highest mean values of pre-dorsal width (PDW) were also found in RC crossbreds during the whole study periods, and the differences of PDW between RC crossbreds and RR purebreds were significantly on the day 90, 152 and 213 (P < 0.05). Significant differences in body weight during the whole grow-out stage except for 92 days were found between two reciprocal crosses (RC and CR), and the yields of RC crossbreds were always higher than those of CR crossbreds.

The absolute growth rates (AGR) of the four groups were the lowest during the period from day 91 to 123, and the highest value of AGR in RC crossbreds and RR purebreds (3.43 and 2.69 g d^−1^, respectively) were recorded in the period from day 214 to 709. The AGR in RC crossbreds were always higher than those of CC purebreds, RR purebreds and CR crossbreds during the period from day 182 to 709 (P < 0.05). The AGR in RC crossbreds was 2.06 times higher than that of RR purebreds, 4.05 times higher than that of CR crossbreds, and 5.42 times higher than that of CC purebreds during the period from day 182 to 213.

### Growth traits and survival under communal rearing condition

Growth traits and survival of the reciprocal cross lines and their parental lines under communal rearing condition in ponds were presented in Table 2. At 314 days old, the mean body weight in RC crossbreds (467.8 ± 110.15 g) was greater than that in RR purebreds (457.8 ± 102.6 g) and CC purebreds (373.4 ± 65.79 g). Furthermore, the standard length, AGR, pre-dorsal width and pre-dorsal height in RC crossbreds were always significantly greater than those in CC purebreds. As the growth rates were considered, the order of the three groups was RC > RR > CC, but no significant difference was detected between RC crossbreds and RR purebreds. In addition, after rearing in the ponds for 334 days, the survival of RC crossbreds (100%) was significantly greater than that of other three groups CC purebreds (92.9%) and RR purebreds (85.7%) (P < 0.05). At 709 days old, the mean body weight in RC crossbreds (2203.0 ± 294.9 g) was significantly greater than that in RR purebreds (1838.0 ± 458.5 g) and CC purebreds (1341.2 ± 350.6 g). Furthermore, the mean values of PDH, PDW and AGR in RC crossbreds were also significantly higher than those in RR purebreds and CC purebreds (P < 0.05). The survival of RC crossbreds (98.5%) was also significant greater than that in RR purebreds (81.4%) and CC purebreds (90.7%) (P < 0.05) (Table 2).

### Heterosis

Table 3 showed mid-parent heterosis (*H*_*M*_), over-CyCa nucleuocytoplasmic hybrid heterosis (*H*_*C*_) and over-Russian scattered mirror carp heterosis (*H*_*R*_) for RC crossbreds under solitary rearing condition from day 90 to 709. The estimates of heterosis for body weight, PDW and PDH were all positive during the whole feeding period. Furthermore, *H*_*M*_ for body weight increased continuously from 8.32% to 46.57% as the feeding time went on. And *H*_*M*_ for AGR (10.12–198.67%) were also positive in different feeding periods except for day 153–181 (−5.46%). For RC crossbreds, both *H*_*M*_ and *H*_*C*_ for standard length were both positive during the whole feeding period. Notable negative *H*_*R*_ was observed for the total length and standard length during the whole grow-out stage.

Heterosis in CR crossbreds under solitary rearing condition from day 90 to 709 was shown in Table 3. *H*_*C*_ for body weight were always positive at different periods, while *H*_*M*_ and *H*_*R*_ for body weight were only positive at day 90 and 709. During the whole feeding periods, *H*_*M*_ and *H*_*R*_ for standard length of CR crossbreds were positive but the values were lower than those of RC crossbreds. *H*_*M*_ and *H*_*R*_ for pre-dorsal width and pre-dorsal height were only positive at day 90 and day 152, but were negative at other feeding periods.

Heterosis for growth traits and survival in RC crossbreds under communal rearing condition were shown in Table 4. For one-year-old RC crossbreds (334 days), the values of *H*_*M*_, *H*_*C*_ and *H*_*R*_ for body weight, absolute growth rates, pre-dorsal width, pre-dorsal height and survival were all positive at the end of feeding periods. After 709 days culturing, the heterosis value for body weight was higher compared to the heterosis in one-year-old RC crossbreds, where the values of *H*_*M*_, *H*_*C*_ and *H*_*R*_ for body weight were up to 38.59%, 19.86% and 64.26%, respectively.

### Correlation analysis among growth-related traits

As shown in Table 5, the correlation between the three growth-related traits (standard length, pre-dorsal length and pre-dorsal width) and body weight were estimated. A significantly positive correlation was observed between body weight and each of the other three morphological traits (P < 0.05), with the Rw values ranging from 0.39 (between pre-dorsal width and body weight for RR purebreds at 123 days old) to 0.96 (between pre-dorsal height and body weight for RC crossbreds at 123 days old) in the three carp varieties during the period from 90 to 334 days old. For the RC crossbreds, both standard length and pre-dorsal length showed significant effects on body weight directly (P < 0.05) from 90 to 334 days old. Before 213 days old, pre-dorsal width was the third leading factor affecting body weight, with the lowest indirect effects from 0.05 to 0.31, whereas pre-dorsal width display higher direct effects (Pi = 0.34) than pre-dorsal height on body weight at 334 days old (P < 0.05). Similar results were also found in both RR purebreds and CC purebreds, where the standard length showed the largest effect on body weight, whereas the pre-dorsal width performed the low indirect effect on the body weight.

## Discussion

As one of the most representative carp species, common carp had a valuable and global production, and the importance of *C. carpio* has been increasing over the past decade. Moreover, the species were also widely cultured as an ornamental fish because of its various color and scale patterns^18^,^19^. In this study, we found that the CyCa nucleocytoplasmic hybrid fish was red color with full scale, the mirror carp was grey color with scattered scale, whereas their hybrid was grey color with full scale. These results demonstrated that the grey color dominated to red color, and the full-scale pattern dominated to scattered scale pattern in common carp. Furthermore, our results also indicated that the CC purebreds, RR purebreds and RC crossbreds were three great materials for the studies of the fish color and scale pattern traits. Recently, Rohner *et al*.^20^ demonstrated that scales loss in mirror carp was caused by loss of function mutations in the gene coding for a receptor of the FGF growth factor named FGFR1a1. In addition, more and more studies tried to reveal the molecular mechanism of fish body color formation^21^,^22^,^23^. Using the CC purebreds, RR purebreds and RC crossbreds as the materials, we also tried to reveal the genetic mechanisms of fish body color and scale pattern, the detail results will be presented in the other papers.

For the growth and survival traits, we investigated growth performances of the purebreds and crossbreds within two different rearing environments, solitary and communal rearing conditions. In solitary rearing condition, the mean values of body weight and AGR in RC crossbreds were higher compared to those in their parents, the CC purebreds and RR purebreds. These results indicated great growth performances of the RC crossbreds to thrive in solitary rearing system. In communal rearing condition, the growth traits in RC crossbreds were significantly higher than those in RR purebreds at 709 days old, and the survival in RC crossbreds was significantly higher than that in RR purebreds. The income of a commercial farmer is determined by the total yield of the carp, which is the combined effect of survival rate and growth rate. With respect to yield, the RC crossbreds will perform quite great compared to the RR purebreds because they have quite high survival rate in the pond. These results, taken together, demonstrated that the RC crossbreds have higher yield than their parents in both solitary and communal rearing system. Basing on the great performances in growth traits and survival, the RC crossbreds will be a potential varieties for commercial production and further selective breeding.

The RC crossbred line displayed positive mid-parent heterosis for all growth traits and survival in this study. Similar results have been reported in other common carp species^24^,^25^. Crosses between genetically differentiated subpopulations are expected to increase heterozygosity, reduce effects of recessive lethal genes and enhance fitness, resulting in heterosis or hybrid vigor^26^. In the present study, as the paternal line, CyCa nucleocytoplamic hybrid fish was the nuclear-cytoplasmic hybridized fish, where the blastulae nucleus of Hebao red carp (*Cyprinus carpio var. wuyuanesis*) was transplanted into the cytoplasm of crucian carp (*Carassius auratus*)^15^,^17^. The maternal line mirror carp was the typical European carp species (*Cyprinus carpio carpio*), which have significant genetic divergence from the Asian carp species (*Cyprinus carpio haematopterus*)^27^,^28^. In our previous study, we also observed that the two parental lines had significant genetic divergence by using the mitochondrial genome sequences^29^,^30^,^31^. These results suggested that the heterosis in growth trait and survival might owe to the genetic divergence between mirror carp and CyCa nucleocytoplasmic hybrid fish. Similar results were also reported in other aquaculture species. Such as, Wang and Xia^32^ showed tendency for positive relationship between heterosis in growth and genetic distances of two interspecific hybrids and one intraspecific crosses of fish. Koolboon *et al*.^8^ reported significant correlations between genetic distance and heterosis in catfish.

In contrast to RC crossbreds, the CR crossbreds performed a poor heterosis in body weight and AGR. Moreover, crosses where Russian scattered mirror carp was used as mother (RC and RR) achieved the greatest performances for all growth traits during the whole grow-out stage. These results, taken together, confirmed the remarkable maternal effects for body weight. Thus, the differences in growth traits between RC crossbreds and CR crossbreds, especially important for RC hybrids, should also be partially attributed to mothering ability from mirror carp. Actually, the maternal influence for growth has also been widely reported in other species^33^. These results, taken together, suggested that both heterosis and maternal effect contributed to the great performances in growth traits and survival in RC crossbreds.

So far, more statistical methods have been employed to estimate the relationships among economically important traits, such as correlations analysis, path analysis and regression analysis. For example, using correlation analyses, Kora *et al*.^34^ reported that standard body length, body weight and body fat content were significantly correlated on cultured red sea bream. In addition, the main factors affecting body weight have also been observed in other fish, such as sea trout^35^, salmon^36^, and red drum^37^. In our present study, standard length and pre-dorsal height showed significant direct effects on body weight (P < 0.05). The correlations coefficient between the two traits and body weight was also high; hence, the standard length and pre-dorsal height could be considered as the main factors affecting the body weight of RC crossbreds, RR purebreds and CC purebreds.

## Materials and Methods

### Fish material and mating design

The CyCa nucleocytoplasmic hybrid fish were cultured in Yangtze River Fisheries Research Institute, Chinese Academy of Fishery Sciences, which was the F3 generation of original CyCa nucleocytoplasmic hybrid fish. The Russian scattered mirror carps were introduced from Russia and kept in Yaowan Experimental Station, Yangtze River Fisheries Research Institute, Chinese Academy of Fishery Sciences. Only mature and healthy male and female fish were used. The mating design was a 2 × 2 diallel cross consisting of two purebred lines [Russian scattered mirror carp (R♀ × R♂, RR) and CyCa nucleocytoplamic hybrid fish (C♀ × C♂, CC)] and their reciprocal crosses lines (R♀ × C♂, RC; and C♀ × R♂, CR) (Fig. 1). In the end of April, when the water temperature has climbed to over 18 °C, six females and six males per subspecies (total 24 parents) were injected with gonadotropin releasing hormone agonist (GnRH-A, 6 mg/kg body weight) and domperidone (DOM, 0.1 mg/kg body weight). Male and female spawning pairs were held in two separated tanks for 10–12 hours. Then the eggs of six females from each subspecies were pooled together and divided into two parts. One part was inseminated by sperm mix from the other subspecies producing two hybrid groups (RC and CR); the rest was inseminated by the sperm mix from the same subspecies, producing two purebred group (RR and CC). The fertilized eggs were spread on fine-mesh nylon net immersed in hatching tanks fitted with a water flow-through system at a water temperature of 18–20 °C for four days. All animal experiments were conducted in accordance with the guidelines and approval of the respective Animal Research and Ethics committees of Huazhong Agricultural University.

### Rearing of fry

The larvae started exogenous feeding on day 3 post-hatching, then two thousand larvae fishes from each cross-type (CC, RR, RC and CR) were stocked in concrete tanks (10 m^2^) for 60 days. There were two replicate tanks for each cross-type. Each tank was aerated using a paddlewheel aerator at night. Natural food (zooplankton), soybean milk, soybean cake and formulated feed were given to fry according to their size.

### Solitary and communal rearing conditions

After 8 weeks culturing, fingerlings (Supplementary Table S1) from two purebreds and crossbreds were stocked into 12 concrete tanks (75 m^2^/each tank) at a density of 200 fish per tanks for solitary rearing experiment, with 3 duplicate tanks for each group. In communal rearing condition, the RC crossbreds were stocked together with two purebred lines RR and CC in the ratios of 100:100:100 in three duplicate ponds (2000 m^2^) for 709 days. There are significant differences in skin color and scale pattern among purebred and crossbred, so it is easy to distinguish RC, RR and CC in the same pond (Supplementary Fig. S1). The stocking density in the ponds was 0.15 common carp per m^2^, together with 1.5 grass carp per m^2^, 0.2 silver carp per m^2^ and 0.2 bighead carp per m^2^. Stocking of silver carp and bighead carp aimed to regulate the water fertility, which was the typical commercial rearing form for carp in China. Fish were hand-fed in tanks, but mechanically fed in ponds with a two-stroke petrol-driven blower connected to a 30 kg vehicle-mounted hopper. Equal portions were offered at each of the two daily meals, and fish were fed by a restricted diet (3–5% body weight) twice daily at am 9:00 and pm 16:00, respectively.

### Measurement of performance and heterosis

In solitary rearing condition, 30 fish in each tank were sampled every four weeks, anaesthetized, bulk weight, counted and returned to the original tanks. The standard length (SL), pre-dorsal height (PDH) and pre-dorsal width (PDW) were measured by using vernier calipers. In communal rearing system, all three hundred fishes in each duplicate group were sampled to determine survival rate, body weight, standard length, pre-dorsal height, pre-dorsal width and absolute growth rate (AGR; g fish^−1^day^−1^) in 334 days and 709 days, respectively. In addition, the skin color and scale types of purebred and hybrid offspring were photographed and numerated during the post-embryonic development (Supplementary Fig. S1).

Heterosis is often expressed as mid-parent heterosis (MPH), comparing the average trait value of the F_1_ hybrid to the average trait value of the parents^38^. Single-parent heterosis (SPH) is defined as the proportional increment in the phenotypic values of single-parent stock caused by crossing. Both MPH and SPH were calculated as follows: MPH = (mean F_1_ − mean both parents)/mean both parents as a percentage^39^; SPH = (mean F_1_ − mean single parent)/mean single parent as a percentage^40^.

### Statistics

One-way analysis of variance (ANOVA) was used to determine the effects of genotype on each of the six performance parameters (survival, body weight, standard body length, pre-dorsal width, pre-dorsal height and AGR) at harvest under solitary and communal rearing conditions, respectively. The correlation among the following traits body weight, body length, pre-dorsal width and pre-dorsal height was calculated, as described by Du and Chen^41^. Homogeneity of variance was assessed using Cochran’s test. If significant differences were found, means were compared using Turkey’s honestly significant differences test (P < 0.05). Statistical analysis of strains was carried out using STATISTICA 6.0.

## Additional Information

**How to cite this article**: Liu, X. *et al*. Performances of the hybrid between CyCa nucleocytplasmic hybrid fish and scattered mirror carp in different culture environments. *Sci. Rep.* **7**, 46329; doi: 10.1038/srep46329 (2017).

**Publisher's note:** Springer Nature remains neutral with regard to jurisdictional claims in published maps and institutional affiliations.

## Supplementary Material

Supplementary Information

## Figures and Tables

**Table 1:** Growth traits (standard length, SL; pre-dorsal height, PDH; pre-dorsal width, PDW; body weight, BW; absolute growth rate, AGR) of four populations reared in solitary rearing condition at different time period (mean ± SD).

	SL(cm)	PDH (cm)	PDW (cm)	BW (g)	AGR(g.d^−1^)
**90d**					**62d–90d**
R♀ × R♂	10.81 ± 0.67^**ab**^	3.93 ± 0.29^**b**^	2.05 ± 1.51^**c**^	41.21 ± 6.6^a^	1.32 ± 0.23^**ab**^
R♀ × C♂	10.72 ± 1.19^**ab**^	4.29 ± 0.4^**a**^	2.22 ± 0.24^**a**^	44.07 ± 12.8^a^	1.48 ± 0.44^**a**^
C♀ × C♂	10.38 ± 0.66^**b**^	4.09 ± 0.29^**c**^	2.16 ± 0.15^**ab**^	40.16 ± 5.6^a^	1.27 ± 0.19^**b**^
C♀ × R♂	10.91 ± 1.0^**a**^	4.12 ± 0.31^**c**^	2.12 ± 0.15^**bc**^	42.9 ± 8.17^a^	1.40 ± 0.28^**ab**^
**123d**					**91d–123d**
R♀ × R♂	13.25 ± 0.96^**a**^	4.77 ± 0.56^**ab**^	2.38 ± 0.13^**ab**^	69.17 ± 16.33^**ab**^	0.90 ± 0.34^**a**^
R♀ × C♂	12.77 ± 0.91^**b**^	4.95 ± 0.44^**a**^	2.42 ± 0.16^**a**^	72.98 ± 18.84^**a**^	0.88 ± 0.57^**ab**^
C♀ × C♂	12.06 ± 0.71^**c**^	4.72 ± 0.35^**b**^	2.41 ± 0.13^**a**^	62.95 ± 9.01^**b**^	0.69 ± 0.27^**bc**^
C♀ × R♂	13.1 ± 0.89^**ab**^	4.66 ± 0.29^**b**^	2.34 ± 0.12^**b**^	63.72 ± 9.61^**b**^	0.63 ± 0.29^**c**^
**152d**					**124d–152d**
R♀ × R♂	15.75 ± 1.19^**a**^	5.55 ± 0.34^**b**^	2.72 ± 0.18^**b**^	110.02 ± 20.73^**a**^	1.41 ± 0.72^**a**^
R♀ × C♂	15.08 ± 1.20^**b**^	5.93 ± 0.41^**a**^	2.93 ± 0.21^**a**^	117.68 ± 20.31^**a**^	1.54 ± 0.70^**a**^
C♀ × C♂	12.82 ± 1.09^**c**^	5.07 ± 0.45^**d**^	2.54 ± 0.22^**c**^	73.58 ± 18.79^**c**^	0.37 ± 0.65^**c**^
C♀ × R♂	14.61 ± 1.20^**b**^	5.32 ± 0.32^**c**^	2.70 ± 0.22^**b**^	91.47 ± 17.75^**b**^	0.96 ± 0.61^**b**^
**181d**					**153d–181d**
R♀ × R♂	16.47 ± 1.25^**a**^	6.18 ± 0.39^**b**^	3.06 ± 0.19^**a**^	148.91 ± 29.09^**a**^	1.34 ± 1.00^a^
R♀ × C♂	15.47 ± 1.50^**b**^	6.46 ± 0.68^**a**^	3.14 ± 0.37^**a**^	149.09 ± 42.2^**a**^	1.08 ± 1.46^ab^
C♀ × C♂	13.43 ± 1.01^**c**^	5.61 ± 0.56^**c**^	3.05 ± 0.28^**a**^	101.13 ± 19.63^**c**^	0.95 ± 0.68^b^
C♀ × R♂	15.5 ± 1.37^**b**^	5.78 ± 0.37^**c**^	2.84 ± 0.27^**b**^	123.08 ± 25.16^**b**^	1.09 ± 0.87^ab^
**213d**					**182d–213d**
R♀ × R♂	17.7 ± 1.34^**a**^	6.35 ± 0.43^**b**^	2.89 ± 0.24^**b**^	164.57 ± 30.03^**b**^	0.49 ± 0.94^**b**^
R♀ × C♂	17.16 ± 1.50^**a**^	6.763 ± 0.46^**a**^	3.19 ± 0.36^**a**^	181.33 ± 35.66^**a**^	1.01 ± 1.11^**a**^
C♀ × C♂	13.68 ± 1.05^**c**^	5.46 ± 0.34^**d**^	2.85 ± 0.24^**b**^	107.07 ± 14.66^**d**^	0.19 ± 0.46^**b**^
C♀ × R♂	16.39 ± 1.47^**b**^	5.75 ± 0.46^**c**^	2.68 ± 0.30^**c**^	131.05 ± 29.94^**c**^	0.25 ± 0.94^**b**^
**709d**					**214–709d**
R♀ × R♂	30.60 ± 3.13^a^	10.92 ± 1.30^b^	6.51 ± 1.29^b^	1503.2 ± 465.7^b^	2.69 ± 0.93^b^
R♀ × C♂	27.02 ± 3.21^a^	13.74 ± 0.84^a^	8.78 ± 0.74^a^	1880.4 ± 378.5^a^	3.43 ± 0.76^a^
C♀ × C♂	21.76 ± 2.14^c^	10.41 ± 0.82^b^	6.68 ± 0.18^b^	1062.7 ± 217.6^d^	1.93 ± 0.43^c^
C♀ × R♂	25.09 ± 2.43^b^	10.16 ± 0.72^b^	6.37 ± 1.12^b^	1364.4 ± 646.2^c^	2.49 ± 1.03^b^

The different letters on the parameters in one column mean significant difference (P < 0.05). R and C represented two parental populations of Russian scattered mirror carp and CyCa nucleocytoplasmic hybrid fish, respectively.

**Table 2:** Growth traits and survival of the purebreds and crossbreds reared in the same big ponds.

	SL (cm)	PDH (cm)	PDW (cm)	BW (g)	AGR (g.d^−1^)	Survival (%)
334 days						
R♀ × C♂	24.89 ± 3.08^a^	8.73 ± 0.72^a^	4.46 ± 0.38^a^	467.8 ± 110.15^a^	1.77 ± 0.42^a^	100 ± 0.00^a^
R♀ × R♂	24.90 ± 1.70^a^	8.74 ± 0.77^a^	4.20 ± 0.38^c^	457.8 ± 102.60^a^	1.72 ± 0.39^a^	85.7 ± 1.72^c^
C♀ × C♂	21.94 ± 1.68^b^	8.48 ± 0.53^b^	4.37 ± 0.36^a^	373.4 ± 65.79^b^	1.40 ± 0.26^b^	92.9 ± 2.01^b^
709 days						
R♀ × C♂	32.29 ± 1.80^a^	16.10 ± 0.89^a^	10.29 ± 0.82^a^	2203.0 ± 294.9^a^	4.63 ± 0.37^a^	98.5 ± 1.65^a^
R♀ × R♂	36.98 ± 2.81^b^	13.34 ± 1.38^b^	7.96 ± 1.01^b^	1838.0 ± 458.5^b^	3.68 ± 0.61^b^	81.4 ± 2.34^c^
C♀ × C♂	26.35 ± 4.12^c^	13.14 ± 1.33^b^	8.44 ± 0.96^b^	1341.2 ± 350.6^c^	2.58 ± 0.46^c^	90.7 ± 1.98^b^

The different letters on the parameters in one column mean significant difference (P < 0.05). R and C represented two parental populations of Russian scattered mirror carp and CyCa nucleocytoplasmic hybrid fish, respectively. SL represented standard length, PDH represented pre-dorsal height, PDW represented pre-dorsal width, BW represented body weight, AGR represented absolute growth rate.

**Table 3:** Mean mid-parent and single parent heterosis of growth traits (standard length, SL; pre-dorsal height, PDH; pre-dorsal width, PDW; head length, BW; absolute growth rate) for RC and CR crossbreds in solitary rearing condition at different time period.

	RC crossbreds					CR crossbreds				
	SL	PDH	PDW	BW	AGR	SL	PDH	PDW	**BW**	**AGR**
90d										
*H*_*M*_	1.18	6.98	5.46	8.32	13.91	2.97	2.74	0.71	5.44	7.75
*H*_*R*_	−0.83	9.16	8.29	6.94	11.72	0.93	4.83	3.41	4.1	5.67
*H*_*C*_	3.28	4.89	2.78	9.74	16.19	5.11	0.73	−1.85	6.82	9.91
123d										
*H*_*M*_	0.91	4.32	1.04	10.48	10.12	3.52	−1.79	−2.3	−3.54	−20.68
*H*_*R*_	−3.62	3.77	1.68	5.51	−2.67	−1.13	−2.31	−1.68	−7.88	−29.89
*H*_*C*_	5.89	4.87	0.41	15.93	26.77	8.62	−1.27	−2.9	1.22	−8.68
152d										
*H*_*M*_	5.57	11.64	11.47	28.19	73.54	2.28	0.1	2.83	−0.36	7.77
*H*_*R*_	−4.25	6.79	7.84	6.96	9.37	−7.24	−4.25	−0.52	−16.86	−32.08
*H*_*C*_	17.63	16.96	15.35	59.93	319.89	13.96	4.87	6.42	24.31	160.76
181d										
*H*_*M*_	3.48	9.58	2.78	19.25	−5.46	3.68	−1.95	−7.04	−1.55	−4.85
*H*_*R*_	−6.07	4.53	2.61	0.12	−19.24	−5.89	−6.47	−7.19	−17.35	−18.72
*H*_*C*_	15.19	15.15	2.95	47.42	14.00	15.41	3.03	−6.89	21.7	14.74
213d										
*H*_*M*_	9.37	14.53	11.15	33.51	198.67	0.35	−0.08	−0.1	−2.39	−0.04
*H*_*R*_	−3.05	6.5	10.38	10.18	106.13	−7.4	−9.45	−7.27	−20.37	−49.08
*H*_*C*_	25.44	23.86	11.93	69.36	441.94	19.81	5.31	−5.96	22.4	33.87
709d										
*H*_*M*_	3.21	28.83	33.13	46.57	48.48	−4.34	−4.97	−3.53	5.97	7.23
*H*_*R*_	−11.69	25.82	34.87	25.09	27.51	−18.01	−6.96	−2.15	−9.23	−7.43
*H*_*C*_	24.17	31.98	31.44	76.95	77.72	15.30	−2.40	−4.64	28.39	29.02

*H*_*M*_ means the mid-parent heterosis, *H*_*C*_ means over-CyCa nucleuocytoplasmic hybrid heterosis, and *H*_*R*_ means over-Russian scattered mirror carp heterosis, respectively. SL, PDH, PDW, BW, and AGR represent standard length, pre-dorsal height, pre-dorsal width, body weight, and absolute growth rate, respectively.

**Table 4:** Mean mid-parent and single-parent heterosis of all growth traits and survival for RC crossbreds in communal rearing condition at different time period (one-year-old and two-year-old).

	SL	PDH	PDW	BW	AGR	SR
334 days						
*H*_*M*_	6.27	1.45	4.01	**12.56**	13.16	**12.00**
*H*_*R*_	−0.04	0.08	6.04	**2.18**	2.55	**16.67**
*H*_*C*_	13.43	3.02	2.06	**25.29**	26.21	**7.69**
709 days						
*H*_*M*_	1.97	21.60	25.49	**38.59**	47.92	**14.47**
*H*_*R*_	−12.68	20.69	29.27	**19.86**	25.82	**21.01**
*H*_*C*_	22.54	22.53	21.92	**64.26**	79.46	**8.60**

*H*_*M*_ means the mid-parent heterosis, *H*_*C*_ means over-CyCa nucleuocytoplasmic hybrid heterosis, and *H*_*R*_ means over-Russian scattered mirror carp heterosis, respectively. SL, PDH, PDW, BW, AGR and SR represent standard length, pre-dorsal height, pre-dorsal width, body weight, absolute growth rate and survival rate, respectively.

**Table 5:** Correlation coefficient (Rw) between three morphological traits (SL, PDH and PDW) and body weight in RC crossbreds, RR purebreds and CC purebreds.

	R × C			R × R			C × C		
	SL	PDH	PDW	SL	PDH	PDW	SL	PDH	PDW
90 days	0.86*	0.91*	0.52*	0.90*	0.91*	0.74*	0.70*	0.75*	0.73*
123 days	0.83*	0.96*	0.68*	0.83*	0.70*	0.39*	0.47*	0.50*	0.49*
152 days	0.88*	0.87*	0.71*	0.96*	0.89*	0.74*	0.93*	0.96*	0.72*
181 days	0.89*	0.93*	0.86*	0.89*	0.92*	0.73*	0.87*	0.82*	0.77*
213 days	0.82*	0.92*	0.58*	0.92*	0.88*	0.66*	0.77*	0.73*	0.52*
334 days	0.83*	0.63*	0.57*	0.91*	0.94*	0.87*	0.77*	0.78*	0.71*

*Means significant difference (P < 0.05). The SL, PDH and PDW represent standard length, pre-dorsal height and pre-dorsal width, respectively.
