# Supplementary material for: Performances of the hybrid between CyCa nucleocytplasmic hybrid fish and scattered mirror carp in different culture environments
Source: Sci Rep. 2017 Apr 12;7:46329. doi: 10.1038/srep46329 (PMC5389345; doi:10.1038/srep46329)
Supplement: Supplementary Information [file srep46329-s1.pdf]

Supplementary Information

Performances of the hybrid between CyCa nucleocytplasmic hybrid fish and scattered mirror carp in different culture environments

Xiangjiang Liu[a#], Hongwei Liang[b#], Zhong Li[b], Yongjun Liang[c], Cuiyun Lu[d], Chitao Li[d], Yumei Chang[d], Guiwei Zou[b*], Guangfu Hu[a*]

a. College of Fisheries, Key Laboratory of Freshwater Animal Breeding, Ministry of Agriculture, Freshwater Aquaculture Collaborative Innovation Center of Hubei Province, Huazhong Agricultural University, Wuhan, 430070, China

b. Yangtze River Fisheries Research Institute, The Chinese Academy of Fisheries Sciences, Wuhan, 430223, China;

c. Beijing Key Laboratory of fishery Biotechnology, Beijing Fisheries Research Institute, Beijing, 100068

d. Key Laboratory of Freshwater Aquatic Biotechnology and Breeding, Ministry of Agriculture, Heilongjiang Fisheries Research Institute, Chinese Academy of Fishery Sciences, Harbin 150070, China.

# These two authors contributed equally to this work.

* Both GF Hu and GW Zou are corresponding authors.

Supplementary Figure 1 Development during the hatching period of embryogenesis and the early larva for RC crossbreds,CC purebreds and RR purebreds.

Supplementary Table 1 Growth traits (standard length, SL; body weight, BW) of four populations at 62 days old (mean ± SD).

Supplementary Fig.1

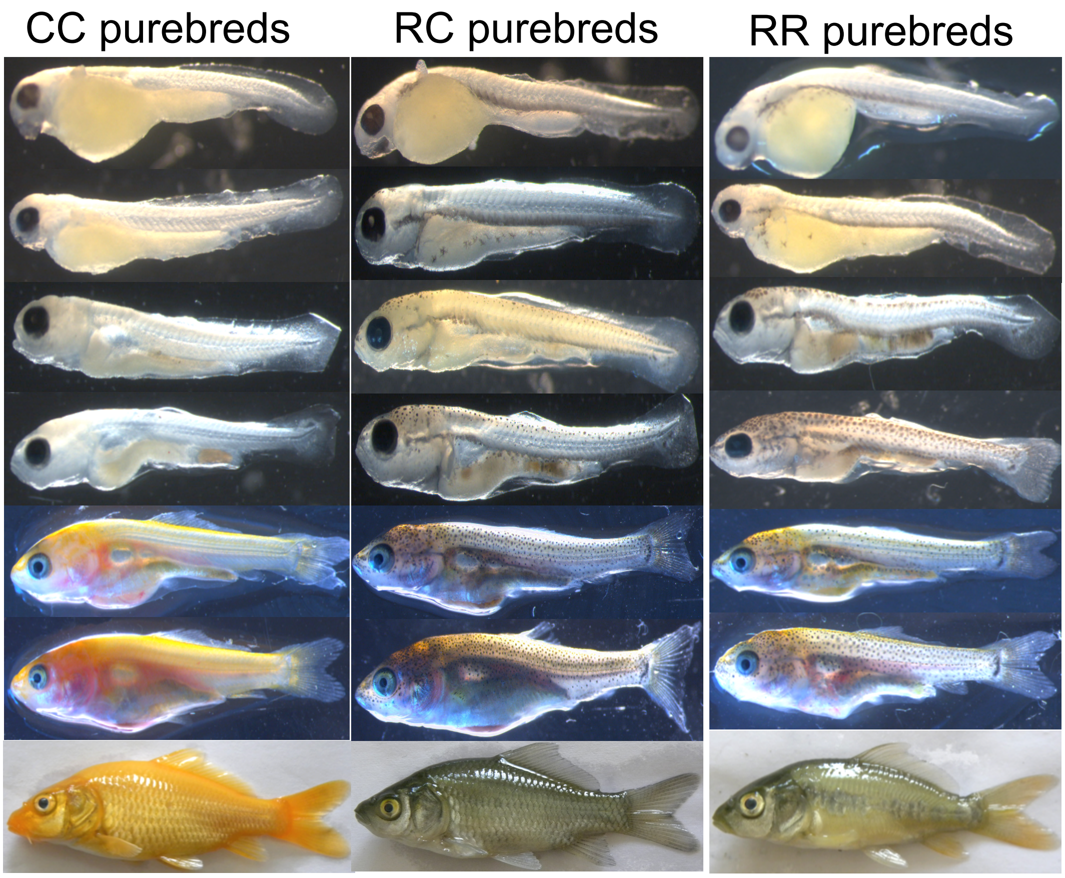

Fig.S1 Development during the hatching period of embryogenesis and the early larva for RC crossbreds, CC purebreds and RR purebreds.

Supplementary Table 1

Table S1

Growth traits (standard length, SL; body weight, BW) of four populations at 62 days old (mean ± SD).

	SL (cm)	BW (g)
R♀× R♂	5.05±0.28[ab]	2.93±0.11[ab]
R♀× C♂	4.72±0.16[b]	2.65±0.09[b]
C♀× C♂	5.22±0.42[a]	3.33±0.21[a]
C♀× R♂	4.81±0.33[b]	2.72±0.12[b]

Noted: The different letters on the parameters in one column mean significant difference ($P<0.05$). R and C represented two parental populations of Russian scattered mirror carp and CyCa nucleocytoplasmic hybrid fish, respectively.
